# Theoretical study of edge states in BC_2_N nanoribbons with zigzag edges

**DOI:** 10.1186/1556-276X-8-341

**Published:** 2013-07-31

**Authors:** Kikuo Harigaya, Tomoaki Kaneko

**Affiliations:** 1 Nanosystem Research Institute, AIST, 1-1-1 Higashi, Tsukuba 305-8565, Japan; 2 Computational Material Science Unit, NIMS, 1-2-1 Sengen, Tsukuba 305-0047, Japan

**Keywords:** Edge states, Flat bands, Zigzag BC_2_N nanoribbons; Tight binding model; First-principles calculations

## Abstract

In this paper, electronic properties of BC_2_N nanoribbons with zigzag edges are studied theoretically using a tight binding model and the first-principles calculations based on the density functional theories. The zigzag BC_2_N nanoribbons have the flat bands when the atoms are arranged as B-C-N-C along the zigzag lines. In this arrangement, the effect of charge transfer is averaged since B and N atoms are doped in same sublattice sites. This effect is important for not only the formation of flat bands but also for the validity of the tight binding model for such system.

## Background

Graphene nanoribbons are finite-width graphene sheets, which are the one of the famous examples of nanocarbon materials [1,2]. The electronic properties of graphene nanoribbons strongly depend on the edge structures. Graphene nanoribbons with zigzag edges have the so-called flat bands at the Fermi level [1,2]. The states corresponding the flat bands are localized at the zigzag edges, i.e., the namely edge states [1,2]. In the honeycomb lattice, there are two inequivalent sites, A and B sublattices. For the formation of edge states, this sublattice structure plays decisive role [1,2].

On the other hand, boron-carbon-nitiride (BCN) materials, such as BCN nanotubes and graphite-like BCN, were synthesized by many groups [3-7]. Quite recently, BCN sheets with BN and graphene domains were synthesized by Ci et al. [8]. Furthermore, a controllability of domain shapes was reported [9]. Fabrication of BCN nanoribbons was expected [10-14]. Therefore, such systems attract considerable interest for application for future electric and optoelectric materials.

Graphite-like BC_2_N sheet is one of the example of BCN, which was synthesized using chemical vapor depositions of boron trichloride, BCl_3_, and acetronitrile, CH_3_CN [15,16]. The stabilities and electronic properties of BC_2_N sheets were investigated by several authors [17-19]. The electronic and magnetic properties of nanoribbons made with BC_2_N sheets were also investigated by several authors [20-24]. The magnetism in BC_2_N nanoribbons is predicted [20,21,23,24]. Xu et al. reported the presence of linear dispersion when atoms are arranged as C-B-N-C in the transverse direction [22].

Previously, the authors reported that the flat bands appear in zigzag BC_2_N nanoribbons where the atoms are arranged as B-C-N-C along the zigzag lines using a tight binding (TB) model [24]. The TB approximation is an efficient method to describe the electronic properties compared with the density functional theories (DFT). In the TB approximation, however, the effect of the charge transfer is absent, resulting in the failure of TB model for B- and N-doped nanocarbon system. The purpose of the paper was to investigate the effect of charge transfer in BC_2_N nanoribbons theoretically.

In this paper, we investigate the electronic properties of BC_2_N nanoribbons with zigzag edges using the TB model and the first-principles calculations based on DFT. The zigzag BC_2_N nanoribbons have the flat bands and edge states when atoms are arranged as B-C-N-C along the zigzag lines. The validity of TB approximation is discussed.

## Methods

**Figure 1:**
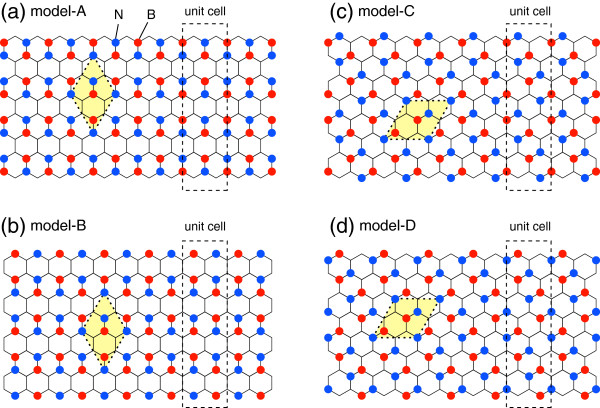
**Schematics of BC2N nanoribbons of the models A (a), B (b), C (c), and D (d).** The red (blue) circles represent B (N) atoms and C atoms are located at the vertices of hexagons. The yellow-shaded dotted lines represent the unit cell of BC_2_N sheet of the model-I introduced in [17]. The unit cell of BC_2_N nanoribbons were indicated by the dashed rectangles.

We shall consider four different structures of BC_2_N nanoribbons with zigzag edges, as shown in Figure 1. In this figure, B (N) atoms are indicated by the red (blue) circles and C atoms are located the empty verticies. Let *N* be the number of zigzag lines of BC_2_N nanoribbons. The dashed rectangles represent the unit cell of BC_2_N nanoribbons. It should be noted that these nanoribbons were made of the same BC_2_N sheet indicated by the yellow-shaded dotted lines in Figure 1 which is the model-I introduced in [17]. The four different models are constructed by cutting the same BC_2_N sheet by changing the cutting positions. In these models, the atoms on the edges are different, as shown in Figure 1. It should be noted that the atoms are arranged as B-C-N-C along zigzag lines in models A and B while do not in models C and D.

We performed the first-principles calculations based on DFT using the local density approximation (LDA) and the projector augmented wave method implemented in VASP code. The cell size in the one-dimensional direction was measured by the lattice constant of BC_2_N sheet, *a* = 4.976 Å, and the ribbons were isolated by vacuum region with about 12 Å in thickness. The outermost atoms are terminated by single H atoms. The geometry was fully optimized when the maximum forces fell down below 10^−3^ eV/Å. The cutoff energy of the plane wave basis set was chosen to be 400 eV, and the *k*-point sampling was chosen to be 12 in the one-dimensional direction. Although we found the finite spin polarization in BC_2_N nanoribbons, we restricted spin unpolarized calculations. The results of spin-polarized band structures will be reported in future publications elsewhere together with other models of BC_2_N nanoribbons.

The Hamiltonian of the system within TB model of *π*-electrons is given by

(1) $$H=\underset{i}{\sum }E_{i}c_{i}^{†}c_{i}-\underset{\langle i,j\rangle }{\sum }t_{i,j}c_{i}^{†}c_{j},$$

where *E*_*i*_ is an energy of *π* electron at the site *i*; $c_{i}^{†}$ and *c*_*i*_ are the creation and annihilation operators of electrons at the lattice site *i*, respectively; 〈*i*,*j*〉 stands for summation over the adjacent atoms; and *t*_*i*,*j*_ is the hopping integral of *π* electrons from *j*th atom to *i*th atom. *E*_*i*_ are the site energies, *E*_B_, *E*_*C*_, and *E*_*N*_, at the B, C, and N sites, respectively. Following the work of Yoshioka et al., we shall assume that the hopping integrals are constant regardless of the atoms, i.e., *t*_*i*,*j*_ ≡ *t*, and *E*_*N*_ = −*E*_B_ and *E*_*C*_ = 0 [25]. For the numerical calculations, we shall choose *E*_B_/*t* = 0.7, 1.0 and 1.3 [24,25].

## Results and discussion

First, we shall discuss the stability of BC_2_N nanoribbons. Calculated formation energies of BC_2_N nanoribbons are summarized in Table 1. Here, the formation energy is defined as

(2) $$E_{\text{form}}=E_{{\text{BC}}_{2}N}-NE_{\text{Gr}}-NE_{\text{BN}}-2E_{H_{2}},$$

**Table 1:** **Calculated formation energies of BC **_**2**_N nanoribbons for **N** **=** **8**

**Model**	**A**	**B**	**C**	**D**
*E*_form_ (eV)	17.173	17.629	15.446	16.532

where $E_{{\text{BC}}_{2}N}$, *E*_Gr_, *E*_BN_, and $E_{H_{2}}$ are total energies of BC_2_N nanoribbons, graphene, boron nitride sheet, and hydrogen molecules, respectively. The model C and D BC_2_N nanoribbons are stable compared with models A and B due to the large number of C-C and B-N bonds. Previously, we considered the BCN nanoribbons where the outermost C atoms were replaced with B and N atoms. In these nanoribbons, H atoms tend to be adsorbed at B atoms [26]. For the model C and D BC_2_N nanoribbons, however, a termination of the outermost B atoms is not energetically favorable compared with a termination of the outermost N atoms. Similar behavior can be found for the zigzag and armchair BN nanoribbons [27]. The outermost B (N) atoms are connected with single N (B) atoms for the model C and D BC_2_N nanoribbons, while the outermost B and N atoms are connected with only C atoms for the previous models’ nanoribbons. Such difference between atomic arrangement should lead different tendency on the enegetics.

**Figure 2:**
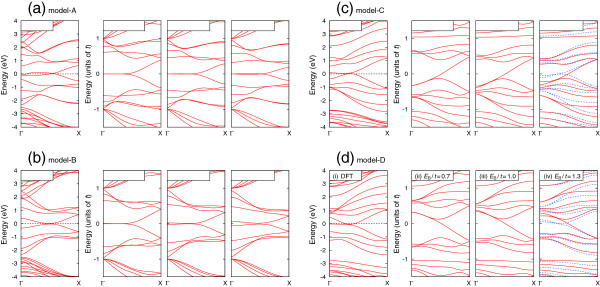
**The band structures of BC**_**2**_**N nanoribbons of the models A (a), B (b), C (c), and D (d) for *N *** **= 8.** In each panel, the result within DFT is shown in (i) and those within TB model are shown in (ii, iii, iv). Note that the center of the energy, *E* = 0, does not mean the Fermi level in models C and D within TB model. In (c - iv) and (d - iv), the improved band structures by adding the extra site energies at the outermost atoms are indicated by the blue dotted lines.

The calculated band structures of BC_2_N nanoribbons for *N* = 8 are summarized in Figure 2. The band structure of the model A nanoribbon within DFT shown in Figure 2a(image i) have nearly degenerate band around the Fermi level. In Figure 2a(images ii, iii, and iv), the band structures of the model A nanoribbons within TB model are shown. We observed that the flat bands and the degree of degeneracy depend on *E*_B_/*t*[24]. The band structure for *E*_B_/*t* = 0.7 has the doubly degenerate flat bands at *E* = 0, but the twofold degeneracy was lifted with increasing *E*_B_[24]. The band structure within DFT resembles to that within TB for *E*_B_/*t* = 1.3 shown in Figure 2a(image iv). The length of the flat bands increase with increasing of *E*_B_, since the shift of the Dirac point of BC_2_N sheet increases [24].

The band structures of the model B nanoribbons also show similar dependence. It should be emphasized that the atoms are arrange as B-C-N-C along the zigzag lines in the model A and B nanoribbons [24]. As reported before [24], we can expect that the bands around the Fermi level would degenerate with increasing of *N*.

In the model C nanoribbons, the band structure within DFT shows the flat bands around the Fermi level, but they are not degenerate. It should be noted that electron-hole symmetry is broken in the model C nanoribbons and atoms are not arranged as B-C-N-C along the zigzag lines. On the other hand, the band structures within TB model do not have the flat bands at *E* = 0. While such prominent bands are not described well, we can see the correspondence between the result within DFT and that of TB model for *E*_B_/*t* = 1.3. Due to the relation *E*_*N*_ = −*E*_B_, the positive energy states of the model C becomes negative in model D, vice versa. Therefore, we can find similar effect to model C in the band structures, i.e., the band structure within TB model of *E*_B_/*t* = 1.3 is similar to that of DFT except around the Fermi level.

We tried to describe the band structure of models C and D using TB model by introducing the extra site energies at the edges. In this study, we added *E*_B_/2 at the outermost N atoms for the model C nanoribbon and −*E*_B_/2 at the outermost B atoms for the model D nanoribbon, because such prescription found to show the relatively good performance. The results for *E*_B_/*t* = 1.3 are shown in Figure 2c(image iv), d(image iv) by the blue dotted lines. The energy bands around *E* = 0 in the vicinity of the Γ are shifted upward (downward) by the prescriptions for model C (D), showing that the band structures became much similar to those within DFT.

**Figure 3:**
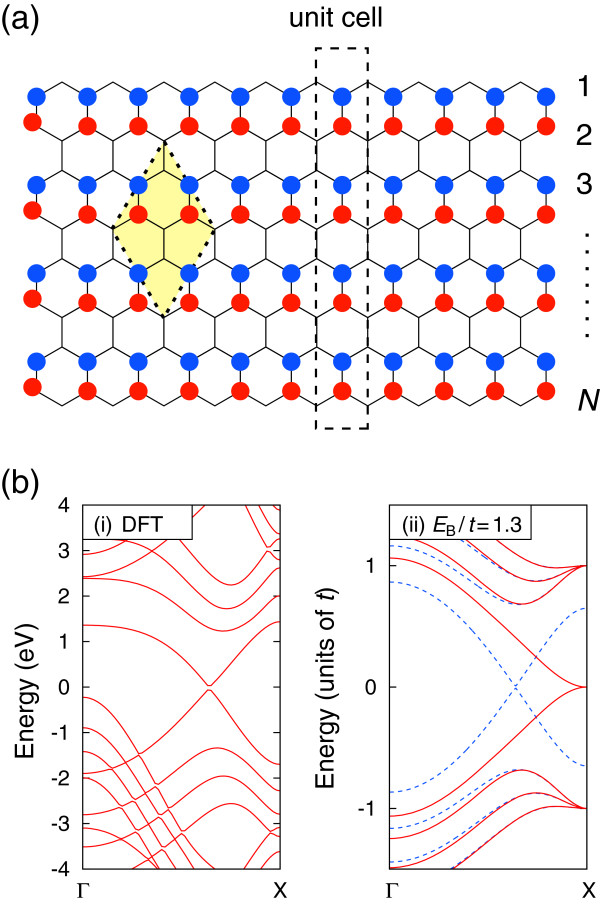
**Model E BC**_**2**_**N nanoribbon.** **(a)** Schematic illustration of model E BC_2_N nanoribbon. **(b)** Calculated band structure of model E BC_2_N nanoribbon shown in **(a)** within DFT (i) and TB model for *E*_B_/*t* = 1.3 (ii).

Previously, Xu et al. reported the band structure within DFT calculations of BC_2_N nanoribbons where the atoms are arranged as C-B-N-C in the transverse direction, as shown in Figure 3a [22]. We shall call these nanoribbons as model E. They obtained the linear dispersion crossing at the Fermi level, as shown in Figure 3b(image i), while the band structure is a semiconducting within TB model, as shown in the red curves of Figure 3b(image ii). In this case, we added *E*_B_/2 (−*E*_B_/2) for the outermost C atoms connected with B (N) atoms. As the results, we could produce the linear dispersion for these nanoribbons as indicated in the blue dashed curves in Figure 3b(image ii). It should be emphasized that all the improved cases have the edge character. Therefore, this prescription works well if the target band keeps the edge character.

**Figure 4:**
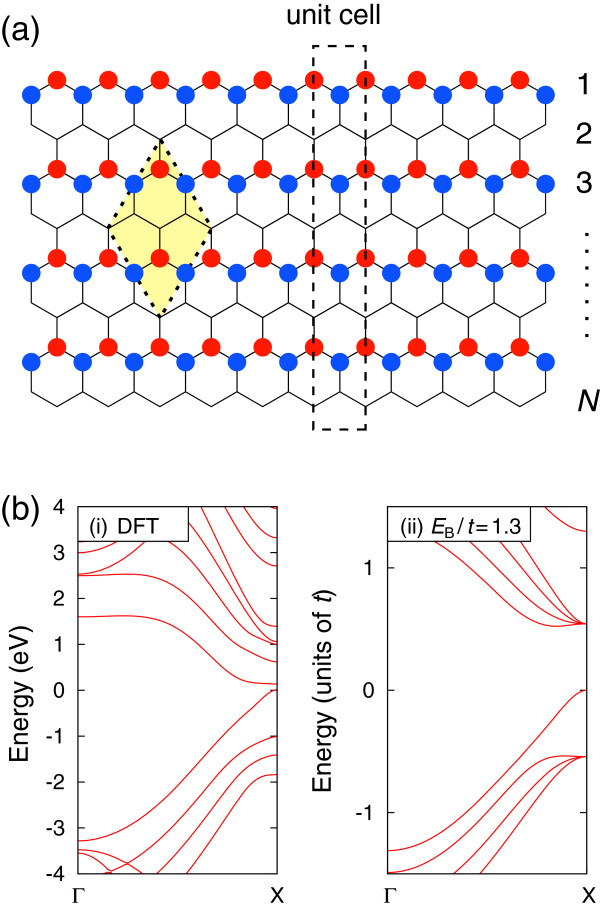
**Model F BC**_**2**_**N nanoribbon.** **(a)** Schematic illustration of model-F BC_2_N nanoribbon. **(b)** Calculated band structure of model F BC_2_N nanoribbon shown in **(a)** within DFT (i) and TB model for *E*_B_/*t* = 1.3 (ii).

The prescription does not work for several BC_2_N nanoribbons. As an example, we shall consider the BC_2_N nanoribbon shown in Figure 4a, which was introducedin [20] as BB-CC model. Here, we shall call the nanoribbons as model F. The model F nanoribbon is made of the other BC_2_N sheet of the model II defined in [17]. Note that the size of unit cell of this nanoribbon is different from those discussed above and the atoms are not arranged as B-C-N-C along zigzag lines in the model F nanoribbons.

Calculated band structures are presented in Figure 4b. As shown in Figure 4b(image ii), the band structure within TB model for *E*_B_/*t* = 1.3 have a finite bandgap which does not decrease with increasing of the ribbon width. On the other hand, the band structure within DFT has a tiny direct bandgap of 0.12 eV at the X point. The decrease of band gap was reported by Lu et al. [20]. It should be noted that we confirmed that the band structure was not improved even if we introduce the site energies at the outermost atoms. Therefore, the arrangement of B-C-N-C along zigzag lines plays a decisive role for the applicability of TB model for BC_2_N nanoribbons.

**Figure 5:**
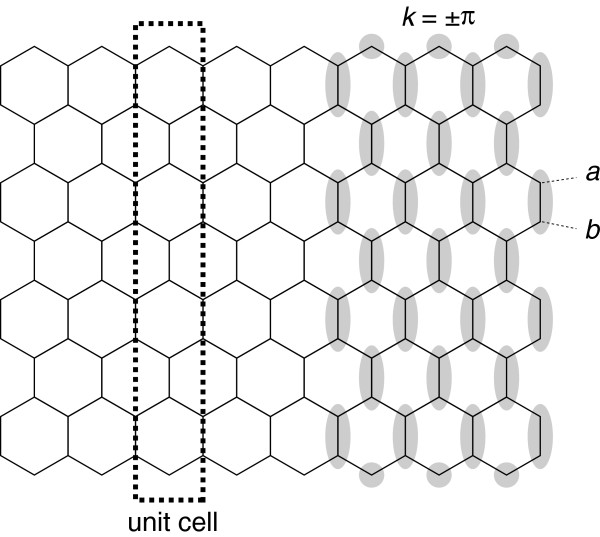
**Schematic illustration of effective decoupling at *k*** **=** **±*π* in zigzag nanoribbons.** Since the hopping integral along the zigzag lines are given by −*te*^±*ik*/2^, the nanoribbons are effectively disconnected as indicated by shaded regions in the right side of figure.

For the zigzag nanoribbons with unit cell being a single primitive cell, the energy at the X point, i.e., *k* = ±*π*, can be solved analytically. Since the matrix elements along the zigzag lines are proportional to −*te*^±*ik*/2^, the hopping along the zigzag lines vanishes at *k* = ±*π* (Figure 5), and the nanoribbons are effectively disconnected as indicated by the shaded region in the right side of Figure 4. Let *E*_*a*_ and *E*_B_ be the site energies at a and b sites shown in Figure 4. In this case, the energies at *k* = ±*π* are given by

(3) $$E=\frac{E_{a}+E_{b}\pm \sqrt{{\left(E_{a}-E_{b}\right)}^{2}+4t^{2}}}{2}.$$

Therefore, the energy bands concentrate on these values at *k* = ±*π* except edge sites, suggesting that the introduction of the edge site energy is not sufficient to improve the description of electronic properties of BC_2_N nanoribbons within TB model.

In the model F nanoribbons, the degeneracy at *k* = *π* within TB model is lifted in the band structure within DFT. The BC_2_N nanoribbons where atoms are arranged as C-B-N-C in the transverse direction do not have such degeneracies. These results indicate that the effect of charge transfer penetrates into interior of nanoribbons, resulting in a formation of transverse gradient of electrostatic potential. In the model C and D nanoribbons, on the other hand, the edge dominant states could not be described within TB calculations. For these nanoribbons, the direction of B-N bonds should play important role. In the BC_2_N sheet shown in Figure 1, the direction of BN dimers is arranged alternately. Then, the formation of transverse gradient of electrostatic potential in the nanoribbons is suppressed due to alternate arrangement of BN dimers in slant angle.

Previously, the authors reported that the arrangement of B-C-N-C along zigzag lines plays a decisive role for the formation of the edge states in zigzag BC_2_Nnanoribbons [24]. This arrangement has other meaning. Within the TB approximation, effect of charge transfer is not described. On the other hand, B (N) atoms act as acceptors (donors) in graphene. Since B and N atoms occupy the same sublattice sites, the effect of charge transfer is canceled when the atoms are arranged as B-C-N-C along zigzag lines. Therefore, TB model is applicable for the zigzag BC_2_N nanoribbons when the atoms are arranged as B-C-N-C along zigzag lines.

## Conclusions

The electronic properties of BC_2_N nanoribbons with zigzag edges have been studied theoretically using the tight binding model and the first-principles calculations. When atoms are arranged as B-C-N-C along the zigzag lines, the zigzag BC_2_N nanoribbons have the flat bands. Then, the tight binding model can become applicable for these systems. In this arrangement, the charge transfer is averaged effectively since B and N atoms are substituted in same sublattice sites, and such effect plays an important role for the formation of the edge states. For the tight binding model, the ratio of the site energies of B atom to the hopping integral is larger than unity. We tried to describe the band structure of BC_2_N nanoribbons where the atoms are not arranged as B-C-N-C along the zigzag lines using the tight binding model by introducing the extra site energies at the outermost atoms, but such method does not work for some BC_2_N nanoribbons. Therefore, study on the electronic properties of BC_2_N nanoribbons should be done within the first-principles calculations.

## Competing interests

Both authors declare that they have no competing interests.

## Authors’ contributions

KH supervised the project and drafted the manuscript. TK carried out the numerical calculations. Both authors read and approved the final manuscript.
